# Study of Wear Phenomenon of a Dental Milling Cutter by Statistical–Mathematical Modeling Based on the Experimental Results

**DOI:** 10.3390/ma15051903

**Published:** 2022-03-03

**Authors:** Filip Ilie, Ioan Alexandru Saracin, Gheorghe Voicu

**Affiliations:** 1 Department of Machine Elements and Tribology, University Politehnica of Bucharest, 060042 Bucharest, Romania; saracinalex@gmail.com; 2 Department of Biotechnical Systems Engineering, University Politehnica of Bucharest, 060042 Bucharest, Romania; ghvoicu_2005@yahoo.com

**Keywords:** statistical–mathematical modeling, dental milling cutters, wear, life cycle, optimization

## Abstract

The wear phenomenon of a dental milling cutter is studied based on experimental results and data and validated by statistical–mathematical modeling. The results of the statistical–mathematical modeling by the interpolation of the experimental results (data) regarding the wear of the dental milling cutter analyzed and obtained in the work process are presented in this paper. These results (data) are important because they lead to polynomial functions which by interpolation approximate very well the dependent parameter, specifically the wear process (mass lost due to dental milling cutter wear, *m**_w_***), considered in the experimental program. The polynomial interpolation functions are valid, only during the experimental testing range of the dental milling cutter, to describe the wear phenomenon; the extrapolations do not lead to satisfactory results. However, by using a controlled interpolation function with an exponential component, the extrapolation of the results is possible. Therefore, the purpose of this paper is the statistical–mathematical modeling by the interpolation of the experimental results of the mass lost due to dental milling cutter wear, *m_w_*, using the deterministic differential model for the work process of it. Thus, interesting conclusions can be drawn relating to the phenomenon. In support of these statements come the results of the statistical–mathematical modeling by the interpolation of the experimental data obtained in the work process of the dental milling cutters, leading to practical applications, such as the extension of the life of dental milling cutter, useful even for its operation optimization; determination of possible criteria for replacing the worn dental milling cutters; the extension of the life of the materials from which dental milling cutters are built; or the provision of ideas for constructive solutions. Based on the modeling results by interpolation, it was found that the dental milling cutter during the milling operation works with high efficiency (mass loss due to wear is very reduced) in the first 11 h of operation, i.e., about a 10% increase in lifetime. After 11 h of operation, mass loss due to wear of the dental milling cutter increases relatively exponentially; thus, it is recommended that, in the normal way, the dental milling cutter be replaced with a new one to ensure high standards of materials processing.

## 1. Introduction

In general, a purely theoretical approach to the phenomenon of contact surfaces wear, with relative motion, is difficult, although not impossible, to model [1,2,3]. In the work process of dental milling cutters, the wear phenomenon is closer to the process of milling by cutting metals, at least until appreciable wear of the active side of the dental milling cutter. The dental milling process is like the process of fine processing of the metals, by milling. Concerns in this regard were presented by Ilie and Ipate [4], where they analyzed the friction state at the interface of two surfaces by measuring the friction force in the polishing/planarization process. In addition, Ilie and Covaliu [5] discussed the antifriction and antiwear behavior of nanoparticle suspensions and presented PM and XPS analysis of the worn surfaces.

In several specialized papers [6,7,8,9], the empirical or theoretical–empirical wear phenomenon is analyzed. Important progress has been made regarding statistical–mathematical modeling of the wear process of a system, in different phases of the life cycle, starting with the process of cutting metals [10,11,12,13,14].

Several mathematical models of analysis of the wear phenomenon of the active side of metalworking instruments exist, presented by Olt et al. [8] and Chinchanikar et al. [11], namely the abrasive model, for steels covered with carbide and low carbon content [15]; the diffusive model, for the material (C-45 steel) of high-velocity milling cutters (HSS) [1]; the Taylor equation model, adapted for estimating tool life [16]; the diffusive–adhesive model, for carbide-coated alloy steels and low carbon content [17]; and the deterministic differential model, for the work process of dental milling cutters [11]. Thus, the deterministic differential model was used in this paper.

Gao et al. [18] studied the wear mechanism of a micromilling cutter with cemented carbide and a 0.6 mm diameter that cut nickel-based superalloy materials. The wear mechanism for the micromilling cutter was established based on the change in the tool diameter and flank width after wear. Then, they set the wear mathematical model for the micromilling cutter.

Complex experimental research on dental milling cutters is still being carried out through advanced studies [19]. Arsecularatne et al. [20] presented interesting experimental results regarding the work process of dental milling cutters. Regarding evaluating dental milling cutters’ wear, the authors used a single parameter given by the values of the pressure from the contact areas between the dental milling cutter and the dental material. In reality, as will be seen, there are several parameters that influence the wear of the dental milling cutters in the working process. A systemic image of the working process of the dental cutters helps to inventory the process parameters, as a result, and of the control of this process, such as the parameters of the dental milling cutter material, the parameters of the dental material which is milled, and the parameters of the operating regime and of the work process (velocity, time, contact force, temperature, and mass lost (the quantitative limit) by the operating dental milling cutters (i.e., they wear)).

Motorcu et al. [21] have investigated flank wear and life of the tool, as well as the effects of cutting parameters in the milling of a superalloy, investigating the effects of cutting parameters (cutting speed, milling direction, coating layer, and the number of inserts of tool holder) on surface roughness. The effects of control factors on the tool life were studied at two cutting velocities and at the lowest value of the average surface roughness parameter (Ra), examining the alternation of flank wear depending on the cutting time. It has been shown that the effect of cutting speed on the tool life was more significant than the effects of the milling type and inserts of the tool holder.

The working process of dental milling cutters is a complex one; many of the basic parameters of the process can be established and quantified only after the mathematical modeling of the phenomenon. Rapid wear of dental milling cutters leads to reduced life, increasing prices for dental operations and for laboratory work. Thus, Klimecka-Tatar [22] investigated the milling tools commonly used in a dental laboratory. In following the obtained experimental results, he found that the milling cutter may become easily slightly damaged during the milling process, the result being the mass loss. In the first 10 h of work, the dental milling cutter operates with high efficiency, and after this period, to ensure high materials processing, the dental milling cutter should be replaced with a new one. On the other hand, Irvinea et al. [23] studied tool wear by identifying the wear mechanism that occurred when carbide tools machined presintered zirconia, and understanding tool wear helps in improving tool design. Tools that have a longer life would allow the end-users (dental technicians) to have less unproductive downtime changing tools. In addition, worn tools produce broken restorations, which is undesirable.

Chinchanikar and Choudhury [11], applying the deterministic differential model for mathematical modeling of the cutting force–time–wear characteristic, studied the interrelationship between the tool wear and cutting forces. The experimental results showed that the cutting force components (feed and radial) are more influenced by tool wear and increase sharply when rapid deterioration of the cutting edges takes place. Hence, the gradual growth of flank wear, rapid deterioration of the cutting edge, and fatal failure due to increased wear rate were correctly sensed by the force signals and proved the existence of a strong interrelationship between the cutting force–wear–time within the different phases of tool lifetime.

Thus, the deterministic differential model was used in this paper to evaluate wear in the work process of the analyzed dental milling cutter. However, none of the critically reviewed studies used the statistical–mathematical modeling of the pressing force–time–rotational velocity–feed (advance)–wear (mass lost due to wear) characteristic, as in the present paper. This modeling allows the extension of the characteristic by including other parameters of the dental cutter that change in the working process, as a result of wear. In addition, the methodology and statistical–mathematical method used for modeling is a novelty in the field, although in the literature there are various models of experimental data, considering one, two, or even three parameters, in other forms and applying other mathematical methods.

Through the wear phenomenon study for the dental milling cutters (of the same type) tested and by the experimental data, it has been shown that practical results can be obtained, such as the extension of lifetime of the cutter (and so of the materials from which the cutter is built) by one hour (compared to 10 h of the literature, i.e., with a percentage increase of 10%) or even working process optimization of the cutter in operation.

Therefore, the purpose of this paper is, through statistical–mathematical modeling and using a deterministic differential model for the work process of dental milling cutters, to lead to practical applications, such as optimizing operation, determining criteria for replacing worn dental milling cutters, increasing the life of cutters and the materials from which they are built, and possibly providing constructive solutions.

The experimental results obtained for the wear of dental milling cutters in the work process were validated by statistical–mathematical modeling by interpolation of these results and led to polynomial functions which approximate very well the dependent parameter (lost mass due to dental milling cutter wear, *m**_w_***). Thus, the results of the modeling showed that in the first 11 h of work, the dental milling cutter works with high efficiency, after which it can be easily decommissioned (optimizing operation, possibly constructive solutions); i.e., it should be replaced with a new one (criterion for replacing of worn dental milling cutter).

## 2. Materials and Method

**Figure 1:**
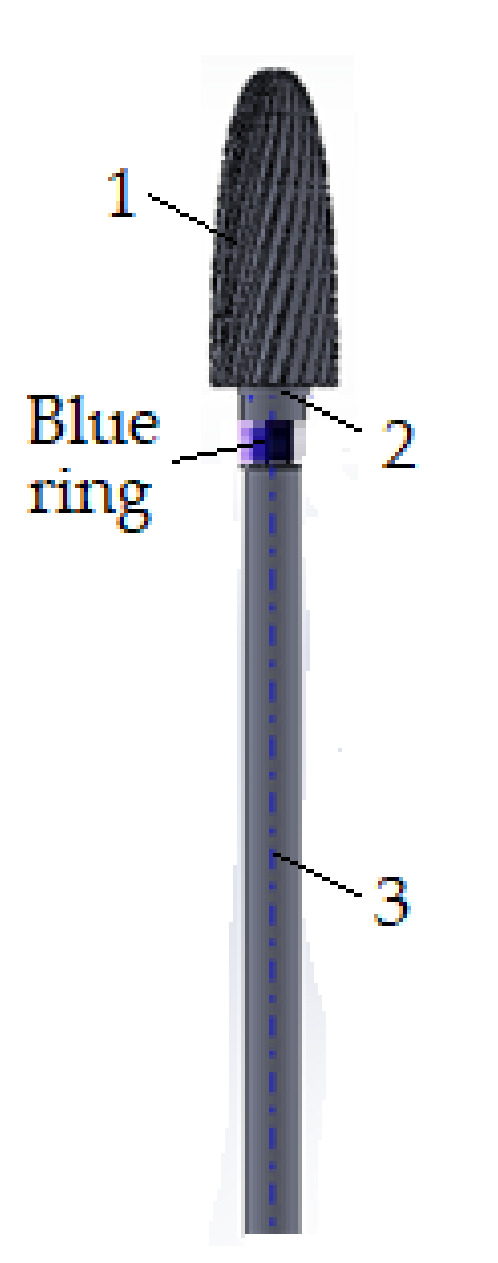
Cylindrical–conical dental milling cutter: 1—the active side of the dental cutter, considered as a standard; 2—cutter neck; 3—cutter leg.

**Figure 2:**
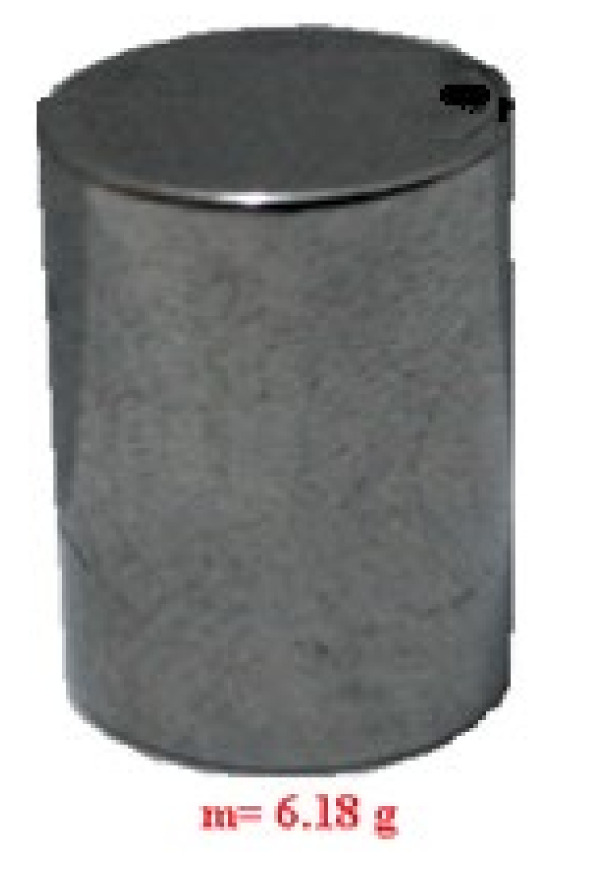
Dental material sample (Ni-Cr alloy).

For the research and the experimental determinations, a cylindrical–conical dental milling cutter was used [24] (type DFS Diadur Quattro 603501 from DFS-Diamon GmbH company, Riedenburg, Germania), shown in Figure 1, and made from a mixture of metal carbides in which predominate by tungsten carbide (CW), together with another carbide of Cr, Mn, Fe, etc., with a medium granulation on the active side. This type of dental milling cutter is used for processing Co-Cr alloys, as well as other semiprecious and precious metals, with a blue ring for identification and works at velocities up to 35,000 rpm. The sample of the dental material used for the experimental tests was made of nickel–chromium (Ni-Cr) alloy because it is a material for dental work in dentistry. The sample, shown in Figure 2, had a cylindrical shape with a diameter of 8.16 mm and a length of 13 mm.

**Figure 3:**
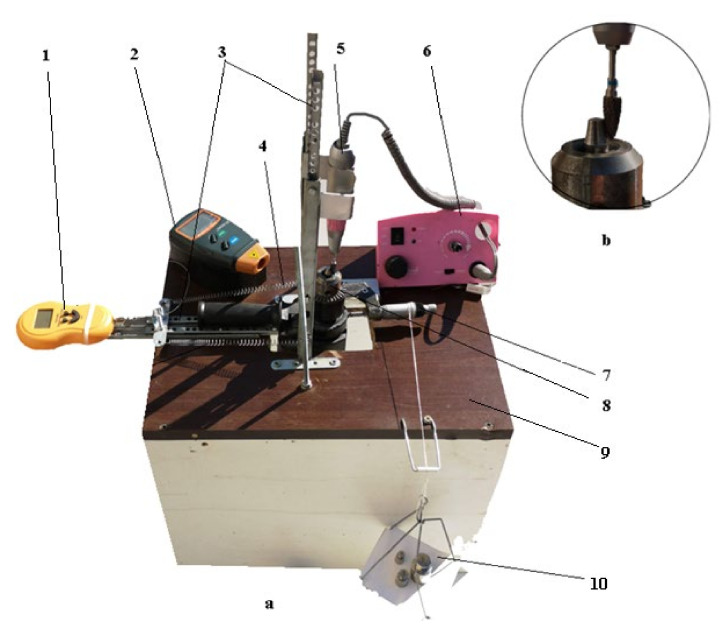
Dental milling cutter test stand: stand overview (**a**); detail view of the material clamp and tooth cutter (**b**): 1—dynamometer, 2—tachometer, 3—mobile support, 4—spring, 5—micromotor, 6—velocity variation, 7—micrometer, 8—chuck, 9—support table, 10—plate.

For the experimental research, an experimental installation made especially for testing, having the components shown in Figure 3, was used. The operation of the installation is considered simple and is described below. The installation consists of a support table (9) on which were placed two movable supports (3), one for the drive micromotor (5) of the dental milling cutter and another for the mandrel micromotor (8) for gripping and driving the milling material. On the same support table (9) were placed a dynamometer (1) for measuring the pressing force between the dental cutter and the milling material, a tachometer (2) for measuring and reading the velocity of the dental cutter and the milling material, a velocity variation (6) for establishing the rotation velocity of the dental milling cutter and its variation, a micrometer 0–25 mm (7) for moving the chuck (establishing the feed rate) with the milling material, a plate with weights (10) for determining the pressing force between the active side of the dental milling cutter and the milling material, and two springs (5) for maintaining the balance between the movement of the chuck with the micrometer screw driven by the plate with the weights set for the pressing force.

Before starting the experimental tests, prior preparation of experimental installation was required. After selecting the type of cutters (see Figure 1) and the dental materials used in the experimental determinations (see Figure 2), these were measured, before, by weighing, and the work process parameters were defined. The list of these parameters is given in Table 1 with the notations and the measurement units.

After establishing the working regimes (rotational velocity, time, pressing force, feed rate) for experimental research, the dental milling cutters were fixed in turn in the support chuck of the micromotor (5) (see Figure 3), which ensures the movement of rotation at the pre-established working regime, and the milling material was mounted in the support chuck (8). Then, the pressing force between the active side of the dental milling cutter and the dental material, the velocity with which the milling is performed, and the time for testing were fixed.

This was followed by the establishment of the rotation velocity of the milling material and its feed rate by placing the weights that ensure the pressing force on the plate (10) and actuating the micrometric screw (see Figure 3), and at the expiration of the time initially set for testing (1, 2, 3, 3.5, and 4 h) for each of the set velocities (7000, 12,000, 20,000, and 35,000 rpm), the dental cutters used for the experimental tests were weighed again.

For the tests, 20 dental milling cutters were used, grouped in four groups of five for each of the velocity regimes, namely 7000, 12,000, 20,000, and 35,000 rpm. In each group, five tests were performed for each of the established working times, namely 1, 2, 3, 3.5, and 4 h. The pressing force (38.88 N, representing the maximum force developed by (obtained on) the experimental installation in Figure 3, specially designed and made for experiments) and the feed rate (1 mm) were kept constant.

The method of experimentation, regarding the wear behavior of dental milling cutters, was preceded by metallographic and chemical analyses, but also by mechanical analyses of the materials used because the wear is a phenomenon that depends on both the material of the dental cutter and the processed dental material, as well as the parameters of the work process (rotational velocity, pressing force, feed, position, etc.), and the time for testing.

## 3. Experimental Results and Discussions

The experimental results are presented and analyzed in this paper, and the object of the analysis constitutes the numerical results, during milling experiments with the dental milling cutters, obtained on the experimental installation from Figure 3. These results prove the time dependence and the rotational velocity of the dental milling cutters, the feed, and the pressing force and the lost material mass from the active side of the dental milling cutters due to wear.

Following the experimental tests and the measured data, the representative values of the working regime parameters (rotational velocity, time, pressing force, feed, initial mass, and the lost mass through wear) were considered, regarding wear behavior of the dental milling cutters, and tested, and the results are presented in Table 2. During the experiments, the values of the main parameters that highlighted the wear of the dental milling cutters, namely the mass of material lost due to wear, *m_w_*, and other significant parameters, were measured at time intervals established.

The pressing force (38.88 N) and the feed (1 mm, as penetration depth (distance), the feed rate being 1 μm/min) were increased constantly and continued to increase (until these values); after 4 h of operation, it was observed that the dental milling cutter no longer advanced in the dental material (the Ni-Cr alloy), so the pressing force and the advance became stationary around these values.

The results obtained from the experimental research on the wear in the work process of the dental milling cutter–dental material pair constitute the data in Table 2. These results are processed and statistically–mathematically modeled in the following, using the deterministic differential model, for the estimation of the lamella wear of the active area of the dental milling cutters in their work process [10]. The analysis of experimental results showed the dependence of the material mass removed by wear, *m_w_*, on the rotation velocity of the dental milling cutter, *ω*; the working time, *t*; the feed, *a*; and pressing force, *F*, of the active side of the dental milling cutters (see Table 2).

It is mentioned that the results regarding the mass lost due to wear of the dental milling cutters (of the same type and model) were obtained by weighing (as specified above). It is observed that the mass lost due to wear of the dental milling cutters increases over time and increases with the rotational velocity of the dental milling cutter, a logical result (to be expected), as will be seen below. On the other hand, it should be noted that the material removal rate increases faster with rotational velocity than with time, which proves that the rotational velocity is the parameter with a higher contribution (influence) to the wear of the dental milling cutter compared to the time parameter. At the same time, the veracity of the experimental results of the mass lost due to wear of the active side of the dental milling cutters results from the fact that they are validated by the statistical–mathematical model developed below, in this section. Moreover, it is important to specify that the rotational velocities selected for experimental tests were not chosen at random, but are frequently used in a dental laboratory and were possible to achieve with the help of the experimental installation in Figure 3. The range of rotational speeds developed by the experimental installation was from 7000 to 35,000 rpm.

The statistical analysis used for processing and modeling of the results obtained experimentally is based on the least-squares method for polynomial interpolation, using Mathcad Prime 6.0 software, a product of Parametric Technology Corporation, Boston, MA, USA. The interpolation results for the parameter measured in experiments, *m_w_*, by the method of the smallest squares are synthesized in Table 3. The table contains the polynomial coefficients with two variables (time, *t*, and rotation velocity of the dental milling cutter, *ω*) and the quadratic average error, which hierarchizes the approximation performances.

For presented results, neither other higher methods of statistical analysis nor interpolation polynomials of degree higher than the third degree can be approached by the used method in this stage. However, the third-degree polynomials proved to be sufficient to describe the wear phenomenon of the active area of dental milling cutters based on the results obtained by modeling, as will be seen, at least at this stage.

Thus, using the general mathematical relation of interpolation polynomials, transcribed in canonical form, based on the data from Table 3 and defined by the following relation [25]:

(1) $$\;\;\;\;\;\;\;\;\;\;\;q\left(t,\omega \right)=\overset{3}{\underset{i=0}{\sum }}\overset{3}{\underset{j=0}{\sum }}c_{ij}t^{i}\omega^{j}\;,$$

which, developed until to the third degree, becomes:

*q(t, ω)* = *c_00_t^0^ω^0^ + c_10_t^1^ω^0^* + *c_20_t^2^ω^0^* + *c_30_t^3^ω^0^* + *c_01_t^0^ω^1^* + *c_02_t^0^ω^2^* + *c_03_t^0^ω^3^*, (2)

where *q* is one of the interpolated parameters (here, the mass lost due to the wear of the dental milling cutter, *m_w_*) expressed by a third-degree polynomial (see Relation (2)), was sufficient to highlight the wear process of the active area of the dental milling cutters. The results of interpolation by the least-squares method for the measured parameter *m_w_* (see Table 1) are given in Table 3 and contain the coefficients of the polynomial with two variables (*t* and *ω* of the dental milling cutters), *c_00_…c_03_*, as well as the average of the quadratic errors, *ε*, which hierarchizes the approximation performances. The polynomial coefficients were calculated with the Mathcad program using the least-squares method and are doubly indexed; thus, the first index (example: *1* of *c_10_*) represents the exponent (power) of the time variable (*t*), and the second index (example: *0* of *c_10_*) represents the exponent (power) of the rotational velocity (ω). For the calculation of the error, *ε*, Relation (3) was used [25]:

(3) $$\varepsilon =100\cdot \frac{\sqrt{{\left(y_{w}-y_{w}\left(x_{w}\right)\right)}^{2}}}{\bar{y}\cdot n},$$

where *y_w_*, *w = 1, …, n* are the experimental data values of the material mass lost due to wear, *m_w_*_(_dependent variable); *x_w_ = t_w_, ω_w_* represent the string values of the independent variables *t* and *ω*; *n* is the number of experimental data (results); and $\bar{y}$ is the average of the values for the experimental data (results) string of the variable, *m_w_*. Applying the mathematical Relation (1) for the measured parameter *m_w_* obtains the following function:

(4) $$\;m_{w}\left(t,\omega \right)=\overset{3}{\underset{i=0}{\sum }}\overset{3}{\underset{j=0}{\sum }}c_{ij}t^{i}\omega^{j}\;,$$

and by replacing the values of the polynomial coefficients (*c_00_...c_03_*) in Table 3, the function (4) becomes:

(5) $$m_{w}\left(t,\omega \right)=6.744\cdot 10^{-5}t-5.699\cdot 10^{-4}\omega -1.028\cdot 10^{-8}t\omega -8.736\cdot 10^{-9}t^{2}\;+6.156\cdot 10^{-7}\omega^{2}+1.762\cdot 10^{-12}t^{2}\omega -1.82\cdot 10^{-12}t\omega^{2}\;+3.37\cdot 10^{-13}t^{3}-1.038\cdot 10^{-10}\omega^{3}$$

**Figure 4:**
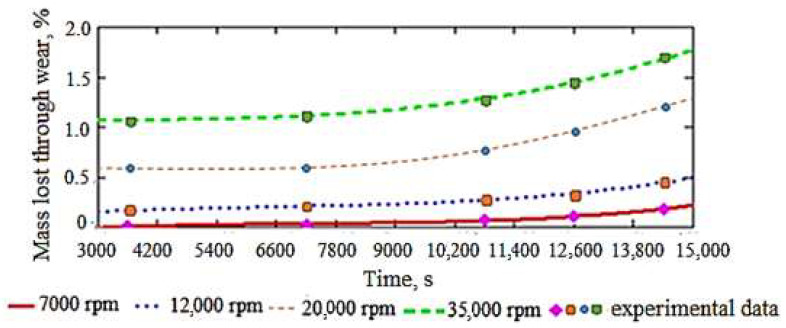
Variation over time of the material mass lost by the active side of a dental milling cutter through wear, described by the interpolation polynomial of third degree at four values of the rotational velocity.

Thus, the variation of the mass of the active area of the dental cutter lost (removed), as a percentage, through wear during the milling process (processing) is presented in Figure 4. The curves in Figure 4 are the graphic representation of the third-degree polynomial equation (according to Relations (4) and (5)) for the rotation velocities of the tested dental milling cutters established by the experimental program. At the same time, the experimental data points corresponding to the interpolation polynomials obtained are marked visibly in Figure 4.

**Figure 5:**
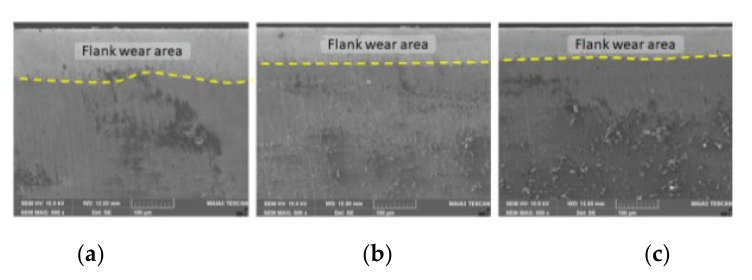
SEM micrographs of the wear of the flanks (lamellae) of the active side of the dental milling cutter at rotational velocity of 20,000 rpm and the feed rate of: (**a**) 0.10 mm/tooth (**b**) 0.30 mm/tooth; (**c**) 0.50 mm/tooth.

From Figure 4, it can be noticed that the mass lost by the dental milling cutters due to wear slightly increases over time and also increases with the rotational velocity of the dental milling cutter (except some little visible minimum points at a lower rotational velocity of 7000 rpm). This confirms that the modeling used obtains results with a similar evolution (although expected) to that of those determined experimentally on the installation in Figure 3 and shown in Table 2; so, it is experimentally validated. Thus, the same is found, both at the experimental and in the theoretical results; i.e., it is justified that both the experiment and the statistical–mathematical model lead to the same knowledge (conclusion). Thus, after 4 h of operation, the mass lost through wear is approximately 0.2% of the initial mass of the dental milling cutter at a velocity of 7000 rpm, 0.4% at a velocity of 12,000 rpm, 0.85% for a velocity of 20,000 rpm, and 1.55% at a velocity of 35,000 rpm, showing a very small percentage increase, which proves the above statement. It is necessary to avoid the extrapolation of these functions for the calculation of mass loss due to wear at rotational velocities of less than 7000 rpm or greater than 35,000 rpm and for time intervals of more than 4 h. The ones stated and observed in Figure 4 are supplemented by the SEM images shown in Figure 5. It is mentioned that SEM images are for an average rotation velocity (20,000 rpm) and increasing feed rate.

**Figure 6:**
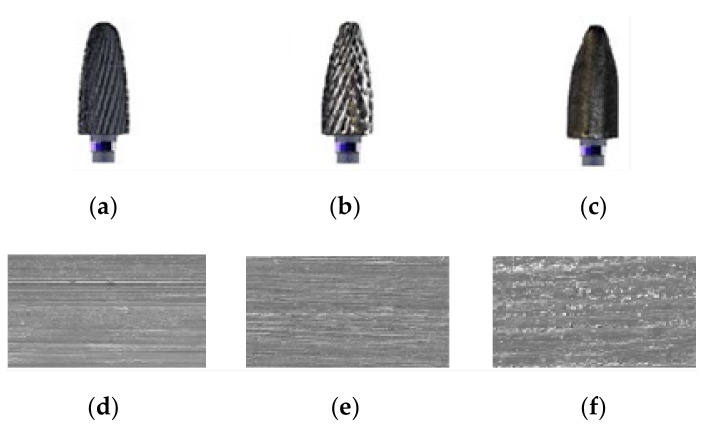
Images of the dental milling cutter wear and of dental material (machined samples): (**a**) standard dental milling cutter after 1 h of operation; (**b**) worn dental milling cutter after 4 h of operation; (**c**) dental milling cutter with the circular crown of the active area, completely worn; (**d**) machined sample of dental material after 1 h of operation; (**e**) machined sample of dental material after 4 h of operation; (**f**) machined sample of dental material after the circular crown of the active area was completely worn. (**d**–**f**) SEM images.

Therefore, it was found that the rotation velocity of the dental milling cutter (*ω*) is the parameter that has a greater influence on its active zone lamella wear compared to the time parameter (*t*), which is consistent with the results of [26,27]. In support of this statement come the images of the dental milling cutter wear in Figure 6, for operation at the rotational velocity of 20,000 rpm, taken at different times (both for dental milling cutter and for material (machining sample) wear): after 1 h of operation (a and d), after 4 h of operation (b and e), and after the circular crown of the active area was completely worn (c and f).

**Figure 7:**
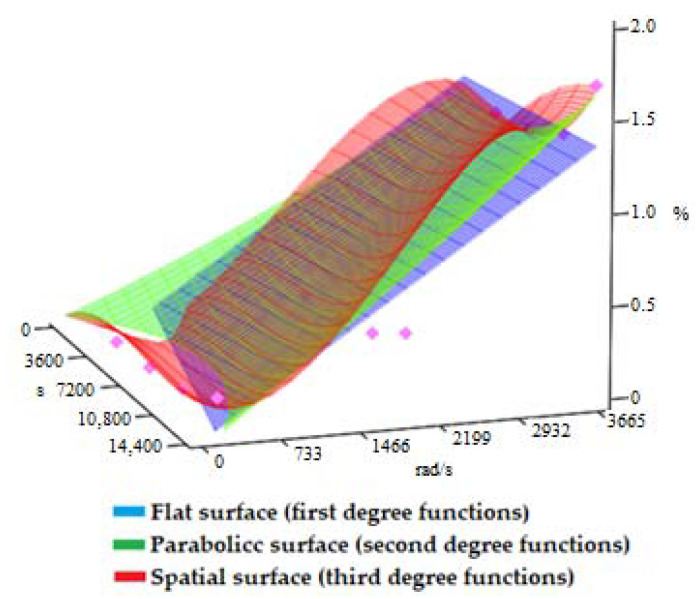
Geometric representation of the interpolation functions of the first degree, second degree, and third degree with two variables, *t* and *ω*, for the mass lost by the active side of the dental milling cutter, *m_w_*, in the work process.

The graphical representation of Figure 7 presents the surfaces geometrically described by first-degree interpolation functions (blue), second-degree interpolation functions (green), and third-degree interpolation functions (red), depending on the two variables: *t,* in s, and *ω*, in rad/s. The shape of the resulting curves is given by the degree of interpolation functions: first degree for flat surfaces, second degree for parabolic surfaces, and third degree for spatial surfaces. In Figure 7, the corresponding experimental data points are also represented as punctual centers in the shape of a rhombus. The interpolation functions were represented by the percentage variation of the mass lost by the active side of the dental milling cutter due to wear, *m_w_*, as dependent on *t* and *ω*.

The interpolation functions for the wear parameter of the active side of the dental milling cutter, *m_w_*, is used to establish the eventually extreme points (of optimal reference) or to estimate any asymptotic behavior with respect to large values as a function of time, *t*, and rotational velocity, ω (i.e., may suggest a quantifiable limit, useful for the decision to replace the dental milling cutter used). After a series of new experiments (experiences), asymptotic or critical points thus determined may be admitted only after experimental validation. It should be noted that the results presented above are specific to the type of dental milling cutters tested. Numerical processing of experimental results (data) by interpolation is a simple operation used generally, especially when no particular aspects are known for the studied phenomenon.

Interpolation polynomials for system parameters (time, *t*; angular velocity, *ω*; and implicitly, angular space, *θ = t·ω*), in this case, are accurate for what we are looking for, but they often do not have a physical interpretation. This means that the polynomial interpolations generally lead to quantitative accumulations and not to qualitative advances, which are assumed for new, additional applications and theoretical explanations. A function based on two hypotheses derived from the experience gained during experimental research is an example of interpolation on the wear processes of cutting or polishing tools:

(1) In the first hypothesis, a function is considered with an exponential component (see Relation (6)), which has an asymptotic behavior for infinite values of the variables ***t*** and ***ω*** (i.e., after reaching a critical value of wear, it models the stopping the lost mass growth) and is canceled at the origin (in the sense that for *t* = 0 or *ω* = 0, the active zone of the dental milling cutter does not change, which was to be expected). This function (6) has an elementary expression and depends on a single variable, *θ* [25]:

(6) $$\varphi \left(\theta ,a,b\right)=a\left(1-e^{-b\cdot \theta }\right),$$

where *a* and *b* are the model parameters; *θ* is the independent variable of function (6), dependent on time, *t*, and rotational velocity, *ω*.

(2) The second hypothesis is the idea of using a single (unique) independent variable, *θ*, which is dependent on the two seemingly independent variables *t* and ω because their product is the angular space (*θ* = *t·ω*) of the dental milling cutter (i.e., the angle rotation traversed in the work process or circumferential friction length).

The hypotheses are in accordance with what was found and observed and the experience gained during the experimental research described above and in correlation with [28]. For the calculation of parameter, *a* from Relation (6), introduction of a new hypothesis was necessary. After a complete operating program, following experimental observations, the dental milling cutter tested no longer cuts or removes particles and chips from the processed dental material. Therefore, there is a limit, where the dental milling cutter no longer removes material (no more works correctly), more precisely, where the lost/removed material mass, both from the active side of the dental milling cutter and from the dental material in the work process, remains approximately constant. Thus, function (6) may be interpreted as having an asymptotic behavior at plus infinity. With these assumptions, the constant parameter, *a* is determined based on the following relation:

(7) $$a=\underset{i=1,\ldots ,n}{\max}m_{w\;i}$$

Then the constant parameter *b* was calculated using the least-squares method applied to the nonlinear function (8), given as follows:

(8) $$\;\varphi \left(b\right)=\overset{n}{\underset{i=1}{\sum }}{\left(\varphi \left(\theta_{i},a,b\right)-m_{w\;i}\right)}^{2},$$

where *θ_i_* has the same meaning and expression but generalized, like *θ*, namely $\theta_{i}=t_{i}\cdot \omega_{i}$, and *t_i_* and *ω_i_*, with *i* = 1, …, *n*, are the data established in advance for experiments.

**Figure 8:**
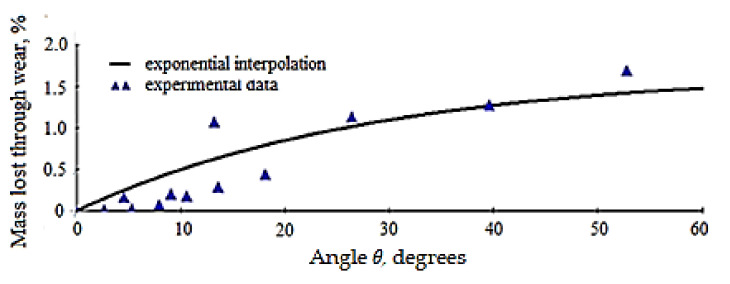
Interpolation function variation (6) for the material mass lost due to wear (*m_w_*) from the active side of the dental milling cutter.

Function (8) is numerically minimized, and for the set of experimental data (results) available in [29], the value of parameter, *b* is *b* = 3.5 ×10^−8^, and in accordance with the experimental data, it follows that, *a* = 1.686. Based on these results, the graphical representation in Figure 8 is obtained, showing the material mass, in percentages (%), lost by the active side of the dental milling cutter, due to wear, depending on *θ* (variable (independent parameter) of the function (6)). The error, *ε*, obtained in this case is *ε* = 14.631%.

It is observed that the degree of scattering of the results is relatively higher for angle values below 20 degrees, which would suggest that the interpolation model does not seem so accurate. We believe that this is due to the cumulative error from the rotation velocity and time because *θ* = *ω t*, which explains the value of the error ε of 14.631%.

By entering *θ = θ (t, ω)* in (6), the interpolation function (9) with two variables is obtained:

(9) $$\;\varphi \left(t,\omega \right)=a\left(1-e^{-b\cdot t\cdot \omega }\right)$$

**Figure 9:**
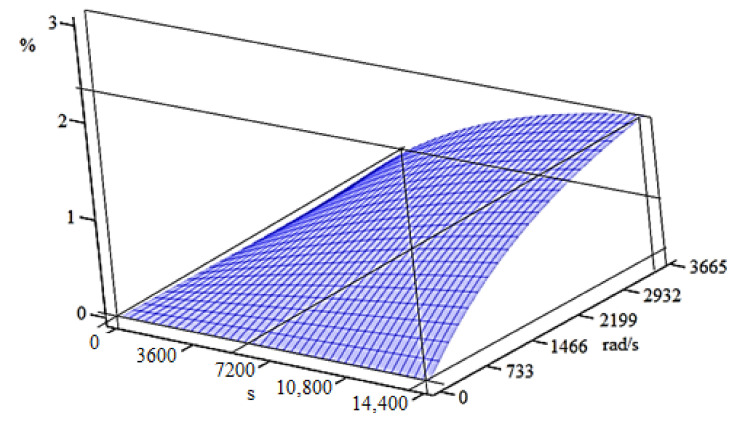
Graphical representation of the interpolation function (9) depending on (*t*, *ω*), i.e., removed/lost material mass, in percentage, due to wear of dental milling cutter.

A graphical representation of the interpolation function (9) depending on the two variables (*t, ω*) is shown in Figure 9. It is noted that this function (9) has an important property, to predict the wear of the active area of the dental milling cutter, namely:

(10) $$\underset{\;\;\;\;\;\;\;\;\;\;\;\;\;\;\;\;\;\;\;\;\;\;\;\;\;\;\;\;\;\;\;\;\;\;\;\;\;\;\;\;\;\;\;\;\;\;\;t\to \infty }{\;\;\;\;\;\;\;\;\;\;\;\;\;\;\;\;\;\;\;\;\;\;\;\;\;\;\;\;\;\;\;\;\;lim}\varphi \left(t,\omega \right)=a>0\;$$

Relation (10) will be used to indicate decommissioning state for the dental milling cutter. For example, when the mass lost due to wear touches a fractional, *μ* from the value of the limit in Relation (10), the dental milling cutter must be decommissioned (declared exhausted). Hence, this state can be written mathematically in the following form:

(11) $$\;\;\;\;\;\;\;\;\;\;\;\;e^{-b\cdot t\cdot \omega }=\mu ,\;\;\mu \in \left(0,\;1\right)$$

From Relation (11), the conventional lifetime, ***t_μ_***, of the active side of the dental milling cutter is obtained:

(12) $$\;\;\;\;\;\;\;\;\;\;\;\;\;\;\;\;\;\;\;\;t_{\mu }=\frac{\ln\frac{1}{1-\mu }}{\omega \cdot b},\;$$

It is observed from Relation (12) that, *t_μ_* (conventional lifetime) is inversely proportional to ***ω*** (rotational velocity of the dental milling cutter). Wear during milling in [14,18] is characterized by a decrease in the diameter of the tool (here, the dental milling cutter) along the length of the active area, which is consistent with what is presented in this paper, on weight loss through wear in the work process of the dental milling cutter. The results obtained based on Relation (12) are given in Table 4.

**Figure 10:**
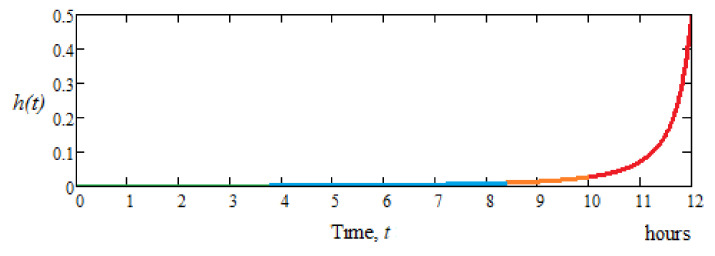
Graph of the instantaneous risk of defecting (defecting probability), *h(t)*, for the set of dental cutters tested.

Knowing the correlation between the wear process and the failure process, as the instantaneous risk of failure (intensity (rate) of defecting), *h(t)*, which expresses the probability that an element (here, the dental milling cutter) will work without defects until time *t*, will be possible in the period following immediately (*Δt* > 0 very small) [30,31]. The graph of the instantaneous risk of defecting (defecting probability), *h(t)*, for the tested dental milling cutters (see Table 2 and Table 4) is shown in Figure 10.

It is observed from Figure 10 that at a time of 11 h, the instantaneous risk of defecting is below 10%. In the 12th hour of operation, the risk of defecting (decommissioning) increases relatively exponentially; i.e., after 11 h of operation, it is normal for the dental milling cutter to be replaced. Moreover, based on the results from Table 4 and Figure 10, it can be observed that the type of dental milling cutter studied works with high efficiency (mass loss due to wear is very reduced) in the first 11 h of operation, i.e., about a 10% increase in lifetime. After 11 h of operation, mass loss due to wear of the dental milling cutter increases rapidly; thus, it is recommended, to ensure high standards of materials milling (processing), that in the normal way the dental milling cutter be replaced with a new one. So, through the wear phenomenon study of the dental milling cutters (of the same type), it has been shown that practical results can be obtained, such as the extension of the lifetime by one hour (compared to 10 h found in the literature, i.e., with a percentage increase of 10%) or even working process optimization of the dental cutters in operation.

## 4. Conclusions

The wear behavior was analyzed through the mass lost by the active side of the dental milling cutters, depending on the process parameters (time, *t*, and rotation velocity, *ω*), and the predominant parameter is the rotation velocity.

The analysis of this phenomenon was performed experimentally and validated by statistical–mathematical modeling based on experimental results obtained from complex research.

Variation over time of the material mass lost by the active side of a dental milling cutter through wear (*m_w_*) is described by interpolation polynomials of third degree, obtaining functions whose variation is dependent on the change in the parameters (time, *t*; rotation velocity, *ω*; feed, *a*; pressing force, *F*) from during the experimental research.

The polynomial interpolation functions allowed the determination of some characteristic points, which represent the extreme values (limits) of wear of the active area of the dental milling cutters but cannot be used for extrapolation.

However, there are functions that at infinity and the origin have controlled behavior and, by interpolation, lead to correct interpretation for the extrapolation of the results (the thing which can be demonstrated only by experimental validation). The example in which the function with the exponential component was used proves this statement.

The independent variables (*t* and ω) of the experimental program can be expressed as one variable, i.e., angular friction distance, *θ* (circumferential friction length).

This proves that although the loss of material occurs over time due to wear, i.e., after the completion of an angular friction distance (friction lengths with variable radius) at the contact of the dental milling cutter and dental material, in the work process not the time which produces the wear, but the actual action.

The problems presented, experienced, and analyzed by modeling regarding dental milling cutters wear also had the purpose of effective practical use (criteria for replacing worn dental milling cutters and increasing their lifetime, the improvement and even the optimization of the operating regime of the cutters and the materials from which they are built, possible constructive solutions).

It was proved that there is a correlation between theory and practice; the results from experiments were validated by statistical–mathematical modeling, leading to the same conclusion.

Based on the results presented in the paper, it was found that dental milling cutters during the milling operation can easily become decommissioned, resulting in mass loss, and in the first 11 h of work, they operate with high efficiency, because the samples of dental material had the highest mass losses. Then, after 11 h of milling work, to ensure high standards of materials processing, the dental milling cutters should be replaced with new ones. However, the applied methodology in the present paper has shown that it is possible to increase the lifetime in this case by about 10%.

It is proposed, for a more complete solution of the problem of wear of dental cutters, to obtain several experimental data and model extensions on other types of dental cutters in order to record the parameters that were not monitored in these first experiments and to suggest future statistical or deterministic models of the phenomenon.

For example, more extensive experiments are recommended, in which the pressing force of the dental milling cutter on the milling material is varied within wider limits. The purpose of this recommendation is to construct a wear forecast function for predicting mass loss through wear with asymptotic variation dependent on the pressing force. In this way, the influence of this important parameter of the work regime will be highlighted, together with the other parameters mentioned in the paper.

The mathematical models that are obtained starting from the structured experimental data and discussed in this paper will be used to investigate the possibilities of improving or optimizing the working regimes of the dental cutters.

## Figures and Tables

**Table 1:** Process parameters.

No. crt.	Parameter	Notation	Measurement Units
1	Time	*t*	s, min, h
2	Rotation velocity of the dental milling cutter	*n (ω)*	rpm (rad/s)
3	Feed (Advance)	*a*	μm/min
4	Pressing force	*F*	N
5	Mass of material by milling wear	*m_w_*	g

**Table 2:** Working regimes for processing the Ni-Cr sample with the dental milling cutter taken as a standard and determining material loss by weighing.

No. crt.	Rotation Velocity (rpm)	Machining Time (h)	Pressure Force (N)	Feed (mm) /Feed Rate (μm/min)	Initial Mass of the Dental Milling Cutter (g)	Total Mass of Material Lost from the Dental Milling Cutter Due to Wear *m_w_* (g)
1	7000	1	38.88	1/1	4.699	0.008
2		2			4.696	0.026
3		3			4.687	0.072
4		3.5			4.677	0.120
5		4			4.659	0.182
6	12,000	1	38.88	1/1	4.667	0.168
7		2			4.660	0.203
8		3			4.645	0.282
9		3.5			4.636	0.628
10		4			4.612	0.690
11	20,000	1	38.88	1/1	4.587	0.570
12		2			4.564	0.686
13		3			4.523	0.804
14		3.5			4.507	1.096
15		4			4.487	1.212
16	35,000	1	38.88	1/1	4.475	1.069
17		2			4.464	1.132
18		3			4.447	1.268
19		3.5			4.387	1.570
20		4			4.364	1.686

**Table 3:** Polynomial coefficients and the error for the parameter, *m_w._*

	*m_w_—*the Interpolation Values of the Degree Polynomial		
Polynomial Coefficients	Degree I	Degree II	Degree III
*_c00_*	−0.315	−3.346 × 10^−3^	0
*_c10_*	1.746 × 10^−5^	−6.150 × 10^−5^	6.744 × 10^−5^
*_c01_*	3.866 × 10^−4^	2.275 × 10^−4^	−5.699 × 10^−4^
*_c11_*	0	1.333 × 10^−8^	−1.028 × 10^−8^
*_c20_*	0	3.792 × 10^−9^	−8.736 × 10^−9^
*_c02_*	0	1.518 × 10^−8^	6.156 × 10^−7^
*_c21_*	0	0	1.762 × 10^−12^
*_c12_*	0	0	−1.820 × 10^−12^
*_c30_*	0	0	3.370 × 10^−13^
*_c03_*	0	0	−1.038 × 10^−10^
ε _, %_	7.828	1.733	0.486

**Table 4:** The calculated lifetime of the active side of dental milling cutters used in experimental research.

No. crt.	Dental Milling Cutter Velocity, rpm		Calculated Lifetime, Hours
1	7000		11.373
2			10.265
3			12.613
4			11.214
5			11.620
6	12,000		6.634
7			5.988
8			7.358
9			6.534
10			6.786
11	20,000		3.848
12			3.411
13			4.098
14			3.684
15			3.884
16	35,000		2.275
17			2.053
18			2.523
19			2.197
20			2.372
Average values of the lifetime calculated, hours		7000	11.417
		12,000	6.660
		20,000	3.785
		35,000	2.284

## Data Availability

Data is contained within the article.

## Nomenclature

*a*	model parameter;
*b*	model parameter;
*c_ij_*	polynomial coefficients;
*e*	number value, e;
*i*	exponent of the time, indices;
*j*	exponent of the rotational velocity, indices;
*m_w_*	mass lost due to wear;
*n*	number of experimental data (results);
*q*	interpolated parameters;
*t*	time, independent variable;
*t_μ_*	conventional lifetime;
*w*	indices;
*x_w_*	string values of the independent variables t and ω;
*y_w_*	experimental data values of the material mass lost due to wear;
$\bar{y}$	average values for the experimental data (results) string.
Greek letters	
*ε*	error;
*θ*	independent variable, angular space, angle;
*μ*	fractional from the mass lost due to wear;
*φ*	function;
*ω*	angle velocity, rotational velocity, independent variable.
